# Pervasive and Persistent Redundancy among Duplicated Genes in Yeast

**DOI:** 10.1371/journal.pgen.1000113

**Published:** 2008-07-04

**Authors:** E. Jedediah Dean, Jerel C. Davis, Ronald W. Davis, Dmitri A. Petrov

**Affiliations:** 1 Stanford Genome Technology Center, Department of Biochemistry, Stanford University, Stanford, California, United States of America; 2 Department of Biological Sciences, Stanford University, Stanford, California, United States of America; University of Oxford, United Kingdom

## Abstract

The loss of functional redundancy is the key process in the evolution of duplicated genes. Here we systematically assess the extent of functional redundancy among a large set of duplicated genes in *Saccharomyces cerevisiae*. We quantify growth rate in rich medium for a large number of *S. cerevisiae* strains that carry single and double deletions of duplicated and singleton genes. We demonstrate that duplicated genes can maintain substantial redundancy for extensive periods of time following duplication (∼100 million years). We find high levels of redundancy among genes duplicated both *via* the whole genome duplication and *via* smaller scale duplications. Further, we see no evidence that two duplicated genes together contribute to fitness in rich medium substantially beyond that of their ancestral progenitor gene. We argue that duplicate genes do not often evolve to behave like singleton genes even after very long periods of time.

## Author Summary

Gene duplication is the primary source of new genes. To persist, duplicated genes must lose some of the original redundancy either by partitioning the ancestral function (subfunctionalization) or by gaining new non-redundant functions (neofunctionalization). The extent to which these processes shape the evolution of duplicated genes over long periods of time is unknown. We investigate these questions experimentally by building strains carrying single and double gene deletions of duplicated genes and measuring their growth rates in rich medium. Using these data, we determine that many duplicated genes are functionally redundant to a substantial degree. We also investigate how often duplicated genes gain new functionality. We demonstrate that the fitness effects of double deletions of duplicate genes are indistinguishable from our best estimate of the fitness effects of deletions of their ancestral singleton genes. We therefore argue that many duplicate genes do not gain substantial new functionality at least in the rich medium. Our results suggest that subfunctionalization does not generally proceed to completion, even after very long periods of time, and that neofunctionalization is either rare or of little consequence, at least under some growth conditions.

## Introduction

**Figure 1:**
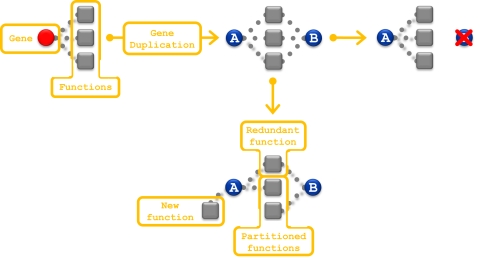
Models for the evolutionary trajectory of a duplicate pair. The squares represent functions and the circles represent genes. Dotted lines connect genes to functions with which they are associated. In this example the ancestral gene was associated with three functions. Following the gene duplication, the duplicate genes (genes A and B) are redundant for all three functions. Because of this redundancy, one copy is likely to be lost by means of an inactivating mutation, as shown in the outcome on the right. The lower outcome illustrates several other possibilities. Here, each duplicate gene has gained non-redundant functionality and now the pair is likely to persist. In this example some of the ancestral function is partitioned between the two duplicates, a redundant function is retained by both, and one of the duplicates (A) has gained new functionality.

Gene duplication is the primary source of new genes [1] and provides essential raw material for the evolution of functional novelty. Upon duplication, the two gene copies are generally assumed to be entirely redundant and either of the gene copies is thus susceptible to loss through inactivating mutations [2]. On this model, in order to persist long-term, the two duplicate copies must lose complete redundancy either by partitioning the ancestral function (subfunctionalization) [3],[4] or by having at least one of the duplicate copies gain a new function (neofunctionalization) [1](Figure 1). Although the loss of redundancy is the key process in the evolution of duplicated genes, it remains poorly characterized. It is not known how quickly complete redundancy is lost, whether it involves both sub- and neofunctionalization, or whether duplicated gene maintain some redundancy over long periods of time.

The extent to which duplicated genes maintain functional redundancy has been assessed by a number of studies. The conclusions of these studies have been equivocal. On one hand, deletions of individual duplicate genes tend to have less severe impacts on growth rate than deletions of individual singleton genes in *S. cerevisiae* [5]. This result has been interpreted as evidence that duplicate genes have higher levels of functional redundancy than singletons. Further supporting this possibility is the observation that deletions of a number of pairs of duplicated genes are synthetically lethal [6],[7] whereas synthetic lethality is extremely rare for double deletions of unrelated singleton genes [8]. This demonstrates that at least some pairs of duplicate genes are redundant for an essential function.

On the other hand, duplicated gene pairs often show substantial divergence in terms of expression patterns [9]–[11], the identity of transcriptional regulators governing their expression [12], and patterns of genetic [7] and protein-protein interactions [13]. Such functional divergence might imply that duplicated genes do become functionally independent to some degree and thus clearly do lose some redundancy. Indeed, many studies implicitly assume that duplicated genes diverge in their functions over time and that sufficiently ancient duplicate genes behave as singletons (*e.g.* [5],[14]). On this assumption, we expect ancient duplicates to show little redundancy above that seen among singleton genes (although, see [15]).

Overall it remains unclear how often and to what extent duplicated genes provide functional redundancy in yeast or in any other organism. Here we directly assay the redundancy among duplicated genes in *S. cerevisiae* at the level of growth rate in rich medium. We define functional redundancy the following way: two genes show functional redundancy if the cost to fitness of losing both genes is more severe than expected under a multiplicative model of interaction [16],[17]. Note that under this definition, two genes carrying out very different biochemical functions might nevertheless appear redundant if their functions are at least partially interchangeable at the level of fitness.

We assess redundancy for a large number of duplicated and singleton genes in yeast. In accordance with expectations derived from previous studies [8], we find no redundancy among our small set 90 pairs of unrelated singleton genes. In contrast, many duplicated gene pairs show substantial redundancy. Specifically, a large number of duplicated gene pairs and none of the singleton gene pairs show synthetic lethality. Duplicated genes appear to remain redundant over very long periods of time, with many retaining substantial redundancy for ∼100 million years. Further, we use these fitness data to investigate the acquisition of new functionality by duplicate genes. Intriguingly, we find that the impacts of the double deletions of duplicate genes are not appreciably greater than the impacts of individual deletions of singleton genes. These data suggest that duplicate genes do not acquire enough new functionality in rich medium, even after long periods of time, to behave similarly to singleton genes.

## Results

### Defining a Set of 289 Duplicate and 90 Singleton Gene Pairs

For simplicity, we limited our investigation to gene families that contain exactly two duplicate genes. We used two strategies to identify such gene families. First, we obtained the list of duplicate gene pairs (Dataset S1) known to originate from a whole genome duplication (WGD) event in the *S. cerevisiae* lineage that took place ∼100 million years ago [18]. These duplicate gene pairs were determined by comparing gene order between duplicated regions of *S. cerevisiae* to gene order in several species that diverged before the WGD [19]–[22]. We removed any pairs of duplicate genes in which either copy had a high degree of identity to a third gene in the genome, (FASTA E-value<0.01) [23],[24]. In total we identified 204 duplicate gene pairs of family size two derived from the WGD event. Second, we identified duplicate pairs resulting from smaller scale duplication (SSD) events. To identify these genes we performed an all-against-all FASTA comparison of the yeast open reading frames (ORFs) available through the *Saccharomyces* Genome Database (SGD) (see Methods) [25], obtained a list of reciprocal best hits, and kept all pairs that showed strong evidence of sequence similarity (FASTA E-value<10^−10^). Again, we removed duplicate gene pairs in which either copy had a high degree of identity to any third gene in the genome (FASTA E-value<0.01), yielding an additional 85 duplicate gene pairs of family size two derived from SSD events.

In addition to the 289 duplicate gene pairs, we defined a set of 90 singleton gene pairs to serve as a control group. Using our FASTA data we first identified a list of 2597 singleton genes, defined here as genes with a low degree of identity to all other gene (E-value>0.01), and that are not present in the WGD list. Using fitness data for a set of strains carrying deletions of individual genes [26], we chose a set of singleton genes such that the distribution of the fitness effects of their deletions approximates that of the chosen duplicates (Figure S1).

### Many Duplicate Gene Pairs Show Evidence for Functional Redundancy

We measured growth rates of strains in rich medium carrying single and double gene deletions of genes within 201 of 289 duplicate gene pairs, as well as within 90 singleton gene pairs. For each of these strains we constructed at least three biological replicates and calculated the mean and standard deviation of the growth rate for each strain. Note that for 32 out of 289 duplicate gene pairs, deletion of one of the two duplicate genes is known to be lethal. We assumed that the double deletions of these pairs are also lethal and thus did not construct strains carrying them. All of these pairs are at least partially non-redundant for essential functionality and it is likely that within this set there are pairs that are fully non-redundant. By excluding them from our analyses we might be upwardly biasing our estimate of the proportion of pairs that show evidence of redundancy.

We elected to stop the strain building after we completed construction of strains to investigate 201 duplicate gene pairs (∼80% of the total). We do not have data for 56 duplicate gene pairs (289 in total minus 32 in which one gene is essential minus 201 for which growth data was obtained leaves 56 unfinished pairs). We know of no systematic bias between those that were completed and those that remain unfinished. For instance, deletions of ribosomal duplicate genes and non-ribosomal duplicate genes exhibit vastly different results, yet we built strains without regard to this functional classification and completed approximately the same proportion of the two sets (86% vs. 77%).

To measure redundancy, we use the fitness of strains with individual gene deletions (*W*_A_ or *W*_B_) and the fitness of strains with the double gene deletion (*W*_AB_). The fitness of a strain is defined as the ratio of the growth rate of the wild type strain in rich medium to the growth rate of the deletion strain in rich medium (see Methods). We define pairs of genes for which *W*_A_ *W*_B_>*W*_AB_ as functionally redundant.

**Figure 2:**
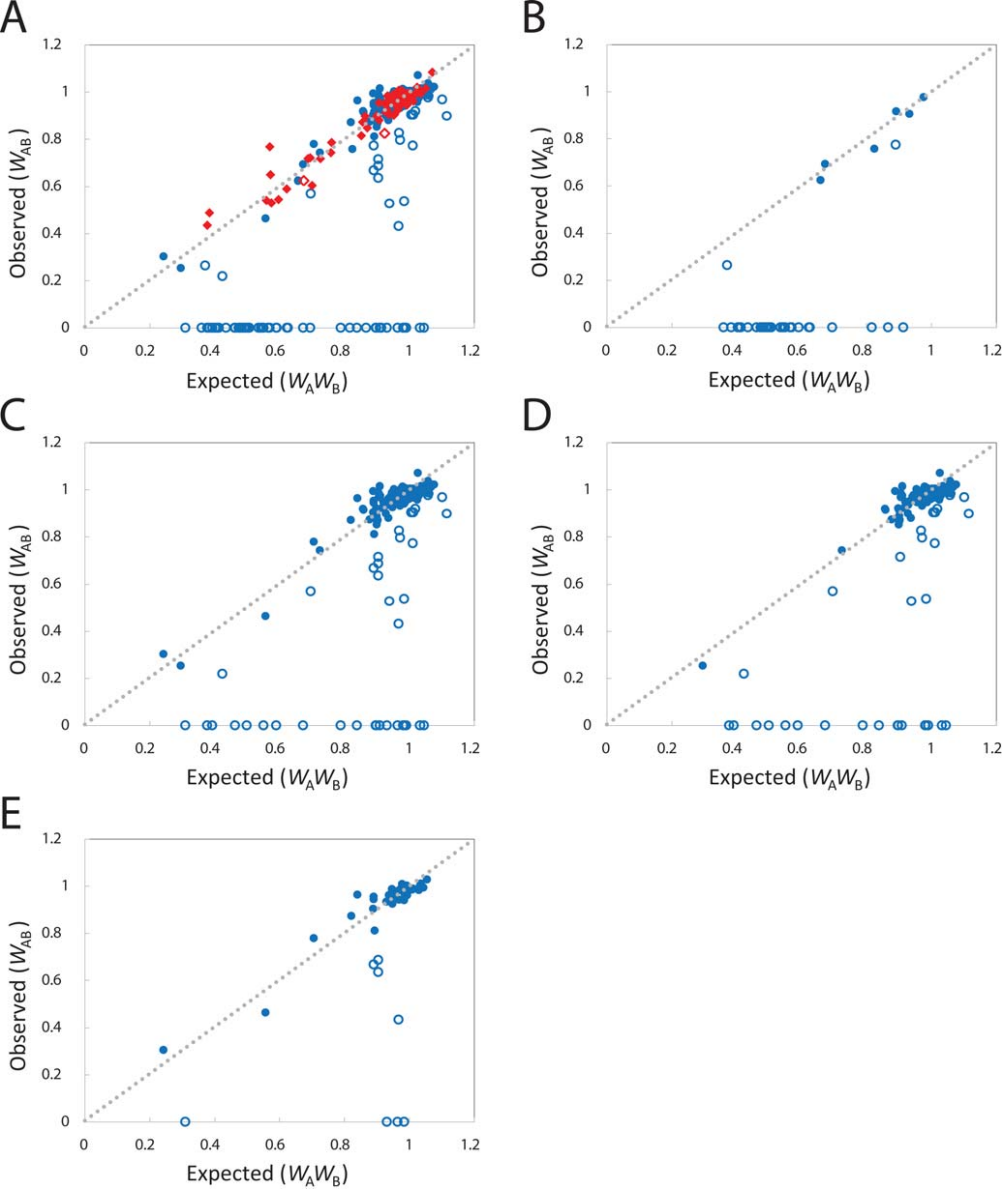
Comparing single gene to double gene deletion fitness values. (A) The observed double gene deletion fitness values (*W*_AB_) are graphed against the expected double gene deletion fitness values (*W*_A_ *W*_B_) for the full set of 90 paired singleton genes (red diamonds) and 201 duplicate gene pairs (blue circles), (B) for ribosomal duplicate gene pairs, (C) for non-ribosomal duplicate gene pairs, (D) for non-ribosomal duplicate gene pairs from the WGD event, and (E) for non-ribosomal duplicate gene pairs from SSD events. The open symbols indicate pairs in which *W*_AB_ is significantly lower than *W*_A_ *W*_B_ (Student's *t* test, *P*<0.025). The grey dotted line indicates a slope of 1 and an intercept of 0 (or *W*_A_ *W*_B_ = *W*_AB_). The pairs in which a single gene deletion is lethal (*W*_A_ or *W*_B_ = 0) were excluded from these figures. Within all of the classes of duplicated genes observed, a significant proportion of pairs show evidence of redundancy. No evidence of redundancy is observed among singleton pairs.

Our results indicate that for the 90 singleton gene pairs, *W*_A_ *W*_B_ approximates *W*_AB_ well (Wilcoxon signed-rank test, *P* = 0.60) (Figure 2A, red diamonds), indicating that there is no evidence for redundancy among unrelated singleton genes. There are only two singleton gene pairs for which the value of *W*_AB_ is significantly lower than the value of *W*_A_ *W*_B_ (Student's *t* test, *P*<0.025) (Figure 2A, open red diamonds). However, this number matches the expected number of false positives given the false discovery rate of 2.5% in our experiment (90×0.025 = 2.25 genes pairs), thus suggesting the absence of any true positives. In contrast, the fitness of double deletions of duplicate genes (*W*_AB_) poorly approximates *W*_A_ *W*_B_ (Wilcoxon signed-rank test, *P* = 10^−12^) (Figure 2A, blue circles), with *W*_AB_ generally being lower than *W*_A_ *W*_B_. Specifically, for 69 out of 201 duplicate gene pairs (34%) *W*_AB_ is significantly lower than *W*_A_ *W*_B_ (Student's *t* test, *P*<0.025) (Figure 2A, open blue circles) including 49 cases (24%) in which the duplicated genes are synthetically lethal (*W*_AB_ = 0). Redundancy appears to be widespread among duplicated genes.

A large proportion (36 of 201) of the duplicate gene pairs in our set encodes protein components of the cytosolic ribosome (*i.e.* the 137 *RPL*, *RPS*, or *RPP* genes) [25]. We tested whether redundancy is confined only to these ribosomal duplicate gene pairs or is prevalent among both ribosomal and non-ribosomal genes. We find that both sets of duplicate gene pairs (Figure 2B and 2C) show significant redundancy, even though ribosomal duplicate gene pairs are more often redundant. Specifically, 39 out of 165 (24%) non-ribosomal and 30 out of 36 (83%) ribosomal gene pairs appear redundant (*χ* ^2^ test, *P* = 10^−11^).

We also tested whether redundancy is observed exclusively within either WGD or SSD pairs. As the vast majority of the ribosomal pairs are derived from the WGD (35 of 36 pairs), we removed these pairs and focused this analysis on the remaining non-ribosomal WGD and non-ribosomal SSD pairs. Of the 165 non-ribosomal duplicate gene pairs, 120 are derived from the WGD event and 45 from SSD events. The gene pairs in both sets (Figure 2D and 2E) are often redundant (31/120 and 8/45 respectively), and have substantial rates of synthetic lethality (4/45 as compared to 17/120). The rates of redundancy and of synthetic lethality between the sets cannot be distinguished (*χ* ^2^ test, *P* = 0.28 and *P* = 0.36 respectively). Admittedly, the power of such a comparison is low and a biologically relevant difference might exist.

### Substantial Levels of Redundancy among Duplicate Gene Pairs

In addition to estimating the proportion of duplicate gene pairs showing redundancy, we also quantified the degree of redundancy (R) within each duplicate gene pair using the expression R = (*W*_A_ *W*_B_−*W*_AB_)/(1−*W*_AB_). R is an estimate of the fitness effect of the redundant function (measured by *W*_A_ *W*_B_−*W*_AB_) compared to the total fitness effect of all of the functions carried out by both duplicated genes (measured by 1−*W*_AB_). Complete redundancy thus corresponds to R = 1 and complete absence of redundancy to R = 0.

**Figure 3:**
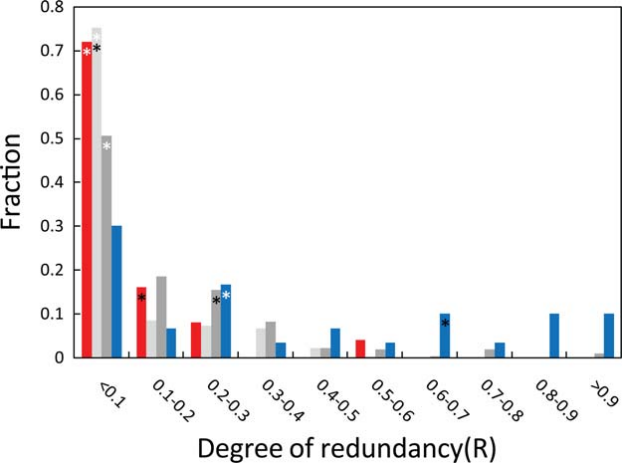
Determining the degree of redundant function within duplicate gene pairs. The histogram shows the degree of redundancy (R = (*W*_A_ *W*_B_−*W*_AB_)/(1−*W*_AB_)) within the set of singleton gene pairs (red), the duplicate gene pairs (blue bars), as well as for the MMS genes when grown in the presence (light grey) and absence (dark grey) of the drug MMS. The white and black asterisks mark the locations of the 50^th^ and 75^th^ percentile values in the figure. The degree of redundancy observed among the duplicate genes is greater than that observed among the singleton gene pairs as well as among the MMS gene pairs grown either in the presence or in the absence of MMS.

Measurements of redundancy using the R statistic were not feasible for two classes of gene pairs. First, the genes comprising the 49 synthetically lethal pairs are at a minimum redundant for an essential function. For cases in which a deletion strain does not show growth, a fitness value is assigned (*W* = 0) rather than measured. Second, for the subset of pairs for which *W*_AB_ is close to 1, the estimate of R becomes overwhelmed by the measurement noise in determining the fitness values. Therefore, we limited this analysis to pairs for which *W*_AB_<0.9 and *W*_AB_≠0. For these 30 duplicate gene pairs, we found that 50% of them (15) have R>0.29 and ∼25% (8) have R>0.69. We compared the distribution of R among duplicate gene pairs to that for a subset of singleton gene pairs selected using the same criteria (*W*_AB_≠0 and *W*_AB_<0.9). The two distributions differ statistically (Mann-Whitney *U* test, *P* = 10^−4^) (Figure 3). Moreover, among these 25 singleton gene pairs, only one pair had R>0.29 and none had R>0.69. As we did not observe a significant number of redundant pairs among the singletons (Figure 2A), we did not expect to see a substantial proportion of the pairs with high R values. Rather, the distribution of R values among the singleton gene pairs gives an approximation of the noise inherent in the measurement. The distributions of R values for the singleton and duplicate gene pairs were considerably different, increasing our confidence that the high R values observed among duplicate gene pairs correspond to high levels of redundancy within these pairs.

### Little Redundancy among Functionally Related Singleton Genes

We considered two ways in which duplicate gene pairs may be functionally redundant. It is possible that redundant duplicates continue to perform some of the same ancestral functions. However, it is also possible that even completely subfunctionalized duplicates, which thus share no ancestral functionality, might still show some functional redundancy simply because they perform related cellular or biochemical roles. For example, duplicated genes that have fully partitioned ancestral function might function in parallel pathways. In such cases our test would likely show redundancy between these genes. In this instance, the signal of redundancy would not derive from retained common ancestral function but rather from the redundancy intrinsic to the network. On the other hand, duplicate gene pairs with fully partitioned ancestral function might operate in the same pathway and therefore show no signal of redundancy.

To assess how much redundancy is expected for genes participating in related cellular roles, we used measurements of the fitness effects of single and double gene deletions of genes important for growth in the presence of the DNA damaging agent MMS [27] (“MMS genes”). Using these data we were able to uncover redundancy (referred to as ‘aggravating genetic interaction’ in [27]) among the MMS genes when the strains were grown in the presence (MMS+) as well as in the absence (MMS−) of MMS. However, using the R statistic we found that the amount of redundancy among the duplicated genes was significantly greater than that observed in the MMS− and MMS+ data (Mann-Whitney *U* test, *P* = 10^−4^ and *P* = 10^−6^) (Figure 3). The R values are higher among the MMS− data as compared to the MMS+ data, however only 17% (55 of 318) of the pairs from the MMS− data have R>0.29 compared to 50% of the duplicate gene pairs. Strikingly, only 3% (9 of 318) of the pairs from MMS− have R>0.69 compared to 25% of the duplicate pairs. These results suggest that a mere similarity of functional roles among pairs of genes is unlikely to be sufficient to generate the substantial functional redundancy seen among the duplicate genes. This analysis strengthens the claim that at least a portion of the apparent functional redundancy among the duplicate genes is due to retention of some of the ancestral functionality by both of the duplicate genes.

Admittedly, the MMS data does not constitute the perfect control for our measure of redundancy among duplicate gene pairs. It might be possible to construct a better control for our global test by matching each duplicate pair to a set of paired singleton genes (Dataset S2) with identical or similar features at the levels of costs to fitness, GO ontology terms, and other functional features, differing only in that these singleton genes do not derive from the same ancestral progenitor. However, it is unlikely that such a test would be preferable to further research on individual redundant gene pairs with the goal of elucidating specific causes of functional redundancy (*e.g.* [28]).

### No Detectable New Functionality Gained by Duplicate Genes

In addition to testing for redundancy, the fitness values of double deletions of duplicate genes can be used to assess how much new functionality has been acquired by duplicate genes. If a duplicate gene pair retains all of the ancestral functionality and gains no additional functionality, then the double gene deletion of the pair of duplicate genes should have the same effect on fitness as the deletion of the ancestral progenitor gene. If, however, one or both members of the duplicate gene pair gain new functionality, the loss of both duplicate genes should generate a more severe cost to fitness than the loss of the ancestral progenitor alone.

The fitness effects of losing the ancestral progenitors are not known. There is reason to believe these ancestral progenitors are a biased subset with respect to their contributions to fitness [29],[30]. Consequently, we developed criteria to choose a proxy set of singleton genes (Dataset S3) that account for the biases among these ancestral progenitors. To do this we made use of a large scale phylogenetic analysis of duplicate genes across 13 yeast species [31]. First, we required the singleton genes to be duplicated in at least one of 12 non-*S. cerevisiae* yeast genomes. Second, the singleton genes in our proxy set may not show evidence of duplication and subsequent gene loss in the *S. cerevisiae* lineage. These criteria yield a list of 305 genes that serve as our proxy set in the analysis of new functionality. In addition, we considered three related proxy sets: (i), all singleton genes, (ii), a set in which the second criterion was eliminated, and (iii), a set in which we added a third criterion requiring the genes to have orthologs in all 12 related yeast species. We used data for strains carrying single gene deletions of the genes in these sets [26] to compare the distributions of fitness values between sets. The proxy set used in the analysis shown below was the most conservative of the three sets (Figure S3) with respect to our conclusions about new functionality gained by duplicate genes.

We determined fitness values for strains carrying single gene deletions of the 305 genes in our proxy set (see Methods). Using these fitness values along with the fitness values for strains carrying double deletions of the duplicate genes (*W*_AB_), we tested whether duplicate genes show evidence of gained functionality. As the distribution of fitness values for ribosomal and non-ribosomal genes is vastly different, the cumulative distribution function of fitness values for our proxy set was adjusted to have the same ratio of ribosomal to non-ribosomal genes as is present within the set of duplicated genes in our study (see Methods).

**Figure 4:**
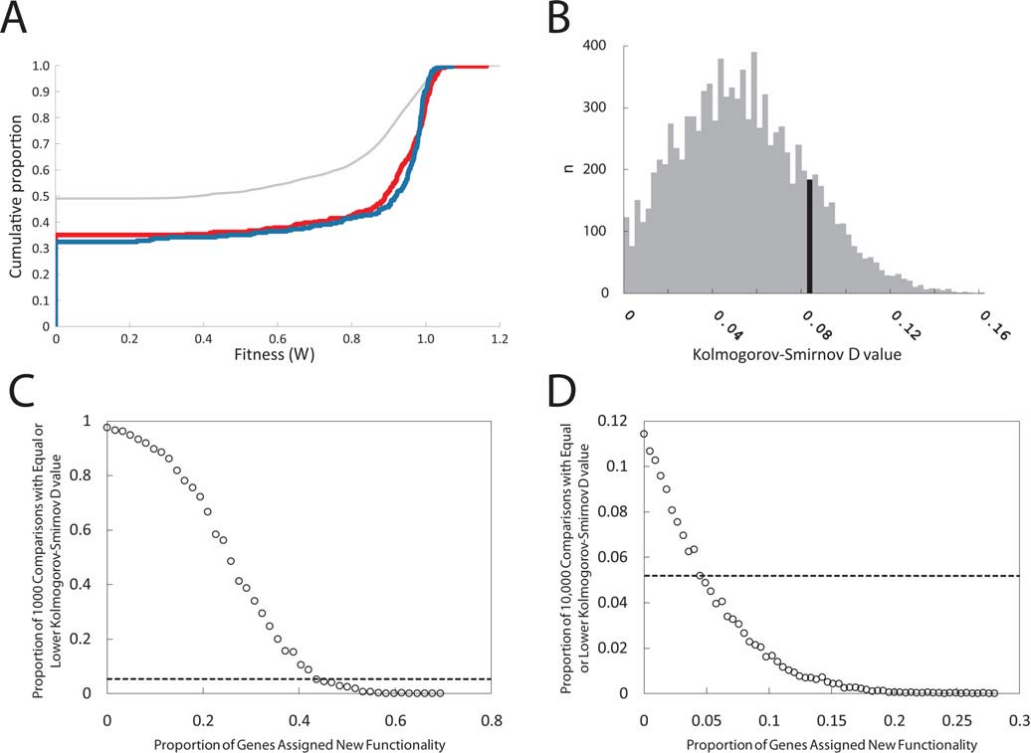
Testing for evidence of gained functionality. (A) The figure shows the empirical cumulative distribution of the fitness values for strains carrying single gene deletions of the singleton genes of the proxy set (red) and strains carrying double gene deletions of duplicate genes (*W*_AB_) (blue). We also include the empirical cumulative distribution of the expected fitness values for all possible double deletions of paired singleton genes from the proxy set (non-ribosomal and ribosomal genes are handled separately, and the proportions are balanced to match that observed among the duplicate gene pairs) (grey). (B) A histogram of 10,000 Kolmogorov-Smirnov *D* values derived from permutations of the data from the proxy set and the set of duplicate genes shown in (A). For the actual distributions of the sets in (A), the Kolmogorov-Smirnov *D* = 0.08. This observed value falls within the black bar on the histogram displaying the 10,000 *D* values derived from permutations of the data in (A). This corresponds to *P* = 0.25 (see Table 1), and indicates that we cannot distinguish these two distributions statistically. (C)(D) Here we assess the sensitivity of our tests for new functionality by building surrogate sets to simulate gained functionality. For each level of gained functionality we constructed 10,000 surrogate sets each simulating a specific amount of gained functionality (see text). We determined the Kolmogorov-Smirnov *D* value between each of the surrogate sets of fitness values and the observed distribution of fitness values for the genes in the proxy set. Here we show how many of the 10,000 comparisons produced a Kolmogorov-Smirnov *D* value that are less than or equal to our observed value of 0.08 (C). We also show how many of the 10,000 comparisons produced Kolmogorov-Smirnov *D* values equal to our observed value of 0 when fitness values above 0.98 are ignored (D).

The key assumption in this analysis is that, with respect to the fitness impacts of their deletions, the extant singletons within the proxy set are similar to the genes that produced the extant duplicate genes. If this assumption is correct, and if the duplicate genes have gained functionality, then we predict that the costs to fitness of deleting duplicate gene pairs (*W*_AB_) should be more severe than the costs to fitness of deleting individual genes within our proxy set. In fact, the distributions of fitness values for the proxy set of genes and for double deletions of duplicate genes (*W*_AB_) cannot be distinguished statistically (Kolmogorov-Smirnov *D* = 0.08, permutation test, *P* = 0.16) (Figure 4A and 4B) (Table 1). Thus, we do not see evidence that pairs of duplicated genes have gained functionality important for the growth of the strains in rich medium.

**Table 1:** Testing for New Functionality.

Comparison	Kolmogorov-Smirnov *D* Value^a^	Permutation Test *P* Value^b^	Kolmogorov-Smirnov *D* Value^a^	Permutation Test *P* Value^b^
	For all fitness values		Excluding fitness values>0.98	
All duplicate pairs vs. Singleton genes in proxy set	0.08	0.16	0	1
Non-ribosomal duplicate pairs vs. Non-ribosomal singleton genes in proxy set	0.10	0.01	0.03	0.53
Non-ribosomal duplicate pairs from the WGD vs. Non-ribosomal singleton genes in proxy set	0.08	0.11	0.002	0.85
Non-ribosomal duplicate pairs from SSD vs. Non-ribosomal singleton genes in proxy set	0.15	0.05	0.10	0.20

a Kolmogorov-Smirnov *D* = max[*Dup*(x)−*Sing*(x)], where *Dup* and *Sing* are the distributions of fitness values for the duplicate and singleton gene sets under test.

b The *P* values described the likelihood of obtaining the *D* value if the two distributions tested were from the same population.

Next, we repeated the test on subsets of our duplicate gene pairs. We compared distributions of *W*_AB_ for non-ribosomal, non-ribosomal WGD, and non-ribosomal SSD duplicate gene pairs to fitness values for strains carrying deletions of the non-ribosomal genes within our proxy set (Table 1). We did not test ribosomal genes as there were too few in our proxy set to make a meaningful comparison. The tests for new functionality among the non-ribosomal duplicate gene pairs and the non-ribosomal SSD pairs did show statistical significance (permutation test, *P* = 0.01 and 0.05). However, closer inspection of the cumulative distributions revealed that this significance is due to the difference between the duplicate genes and proxy set genes near a fitness value of 1. Specifically, when we repeated this analysis ignoring all fitness values greater than 0.98, the significance disappeared (Table 1; *P*>0.05 for all comparisons). Because the fitness values near 1 are strongly affected by noise, we believe that the original significance is due to the differences in the precision with which fitness values were determined for our two sets of strains (*i.e.* for strains carrying single deletions of singleton genes and strains carrying double deletions of duplicate genes). However, even if the difference is biologically genuine, this analysis revealed that the gain of functionality by duplicate genes is minor at best.

Finally, we tested our sensitivity to detect gained functionality. We simulated gained functionality by constructing surrogate sets of 225 fitness values where, in each surrogate set, a specific proportion of the fitness values are derived from the expected fitness values of double deletions of singleton genes. The rest of the values are sampled, with replacement, directly from the distribution of fitness values in the proxy set. The expected fitness values for double deletions of singleton genes were generated by taking the product of two random fitness values sampled, with replacement, from the proxy set. We also required the surrogate sets to maintain the ratio of ribosomal to non-ribosomal fitness values observed in the duplicate set (i.e. 39 to 186). For each specific amount of simulated gained functionality, we built 10,000 replicate surrogate sets each with 225 fitness values (corresponding to the number of double deletions of duplicate genes in our collection). We then measured the Kolmogorov-Smirnov *D* value between each of the surrogate sets of fitness values and the observed distribution of fitness values for the genes in the proxy set. For each level of simulated new functionality we asked how many of the 10,000 comparisons produced a Kolmogorov-Smirnov *D* value that was less than or equal to our observed value of 0.08 (Figure 4C). In addition, we asked how many of the 10,000 comparisons produced Kolmogorov-Smirnov *D* values equal to our observed value of 0 when fitness values above 0.98 are ignored (Figure 4D).

Our sensitivity to new functionality is much higher when fitness values strongly affected by differences in measurement precision (those above 0.98) are ignored (Figure 4D). Among these comparisons, if more than 4% of the fitness values in the surrogate sets correspond to the expected values for double deletions of singleton genes, then the Kolmogorov-Smirnov *D* values are greater than the observed *D* value in over 95% of the 10,000 comparisons (Figure 4D). When we include fitness values greater than 0.98 in this analysis, our test is not longer as sensitive (Figure 4C). It is not until 45% of the fitness values in the surrogate set correspond to the expected values for double deletions of singleton genes that the Kolmogorov-Smirnov *D* value are consistently greater than the observed *D* value (Figure 4C).

For reference, we have included the cumulative distribution function of fitness values for all possible double deletions of singleton genes within our proxy set (corrected for the ratio of non-ribosomal to ribosomal fitness values), as determined by the multiplicative model of interaction (Figure 4C, grey thin line). This distribution represents the expected impact of double deletions of duplicated genes if their combined functional importance was as great as that of two unrelated singletons. We built 10,000 surrogate sets of 225 fitness values from this distribution and found that none had a Kolmogorov-Smirnov *D* value less than the observed Kolmogorov-Smirnov *D* value for the tests of our duplicate gene pairs. In conclusion, our test has substantial power to detect new functionality. This suggests that our conclusion that duplicate genes gain little new functionality in the rich media even after long periods of time is robust.

## Discussion

In this study we systematically quantify functional redundancy for a set of duplicated genes in *S. cerevisiae* that exist in exactly two copies, as well as for a comparable set of paired singleton genes. While we discover that duplicated genes commonly show redundancy, we cannot detect redundancy for any of the singleton pairs. This redundancy appears to be a general property of duplicated genes in our set and is independent of whether they are associated with ribosomal or non-ribosomal functions, or have been generated by WGD or SSD. Even ancient duplicate gene pairs that have been evolving for ∼100 million years often show redundancy.

In many cases, the degree of redundancy exhibited by duplicated genes is substantial. For instance, a quarter of all duplicate genes are redundant for at least one essential function (they are synthetically lethal). Of these more than 30% have an expected fitness value (*W*_A_ *W*_B_) greater than 0.8 indicating that the redundant functionality contributes considerably to fitness. We also estimate that for approximately 50% of the non-synthetically lethal duplicate genes the fitness effect of the redundant functionality is greater than 30% of the total fitness effect of all the functions carried out by both genes (*i.e.* R>0.30).

We considered two potential explanations for the pervasive functional redundancy evident among duplicated genes. First, duplicate genes that have partitioned ancestral functionality might perform similar functions in the organism. It is possible (but not necessarily likely) that any two functionally related genes would show high levels of redundancy. For example, even though genes participating in parallel biochemical pathways should show more redundancy than entirely unrelated genes, genes participating in the same pathway should show lower levels of redundancy than randomly paired genes. Nonetheless, there is some evidence that functionally related genes might show a higher degree of redundancy than unrelated genes. For example, genes that are important for cell survival and growth after treatment with a DNA damaging agent MMS [27] (“MMS genes”) and which thus participate in related cellular roles do show some redundancy (Figure 3). However, the data for the MMS genes grown either in the presence or in the absence of MMS do not show the same degree of redundancy we observe among the duplicate genes. Thus we argue that functional similarity among duplicate genes is unlikely to account completely for the degree of redundancy displayed by the duplicated genes.

The other explanation is that redundant functionality comes from shared ancestral function. Non-redundant functions correspond either to subfunctions that have been partitioned between the two duplicates or to independently acquired new functions. Why would two duplicate genes continue sharing the ancestral function over long periods of evolutionary times (*i.e.* why does subfunctionalization not proceed to completion)? One possibility is that functional similarity within the duplicate pair is maintained by selection for its effect on the level, rate, dynamics, or noisiness of expression [32]–[39]. This might be the case for duplicate genes that encode parts of macromolecular complexes. In this case there could be a need to maintain a stoichiometrically precise balance in gene dosage [32]. This is likely to be the reason for high levels of redundancy shown by duplicated genes that encode components of the ribosome. Indeed, 51.1% of the ribosomal duplicate genes are haploinsufficient [40] indicating that *S. cerevisiae* is sensitive to dosage changes of the ribosomal genes. Also consistent with stoichiometric constraints, most of the protein components of the ribosome are duplicated genes (86.9%), and the vast majority of these duplicated genes derive from the WGD event (92.4%). This is the pattern of observations expected under the stoichiometric explanation as ribosomal genes duplicated one-by-one would have a deleterious effect on fitness. At the same time, following a simultaneous duplication of all the ribosomal genes by means of a WGD event, losing any single gene would have a deleterious effect [32],[41]. This, however, does not provide a full explanation for the evolution of these pairs as it is known that duplicated ribosomal genes also possess some non-redundant functionality [42],[43].

Among the non-ribosomal duplicate gene pairs we do not see the same signs of selection for the stoichiometrically determined gene dosage. Only 1.2% of the non-ribosomal duplicated genes are haploinsufficient, giving little indication of dosage sensitivity for non-ribosomal duplicates. Furthermore, redundancy is common not only for duplicate pairs derived from the WGD but also for those generated by the SSD events. It remains possible that redundancy for non-ribosomal genes is maintained by selection for elevated rates or levels of expression that are not stoichiometrically determined. In addition, having two redundant loci might help buffer against stochastic fluctuations in expression level, as for certain genes such stochastic variability might be deleterious [38],[39]. In some cases, it is possible that even the initial fixation of the duplicated copy was due to the advantageous effect that it immediately had on various properties of gene expression [33],[36]. In such cases, as long as the benefit of these specific properties of gene expression remains, the two duplicates would be maintained in the genome and would retain redundancy.

Finally, it is possible that a portion of the ancestral functionality cannot be partitioned because both duplicate genes might require it in order to perform their non-redundant functions. In this case, mutations that lead to additional subfunctionalization also inevitably inactivate the gene entirely or lead to dominant negative forms of the protein. These and all of the above possibilities are not mutually exclusive and will need to be assessed explicitly in future research.

The other major conclusion of our study is that duplicate genes do not appear to acquire new functionality in rich medium, even after very long periods of evolution (∼100 million years). If duplicated genes have not gained new functionality, we expect strains carrying double gene deletions of duplicate genes to have costs to fitness similar to strains carrying single gene deletions of singleton genes. However, if duplicated genes have gained enough new functionality to behave as two independent singleton genes, then removing a duplicate gene pair should have a cost to fitness comparable to that of removing two singleton genes. Because the ancestral progenitors of the duplicate genes might be a biased subset of genes [29],[30], we developed a proxy set of singleton genes to account for this potential bias. The distribution of fitness values for strains carrying single deletions of singletons in the proxy set is similar to that for strains carrying double deletions of duplicates. Because our test is sufficiently sensitive to detect changes in gained functionality, we conclude that duplicate genes have not gained substantial new functionality in rich medium (Figure S2). Additional work is needed to understand discrepancies between our study and various other predictions of new functionality (*e.g.* [11],[15],[22],[44],[45], although see [46],[47]).

Two alternative explanations might account for the lack of appreciable new functionality. First, our measurements of fitness were carried out exclusively in rich medium (YPD). Although work done in this single condition is sufficient to conclude that duplicate genes are highly dissimilar from singleton genes with regard to contributions to fitness, it does not resolve the question of whether duplicate genes gain new functionality. That is, any new functionality acquired by duplicated genes that is only important under alternative environmental conditions would not be detected using this assay. Indeed, several studies show that duplicated genes are often involved in interacting with the environment and managing stress [36],[48]. Future measurements of the fitness of deletion strains under various environmental conditions should address this possibility.

Second, it is possible that the maintenance of redundancy and the lack of acquisition of new functions are related to each other. In this scenario, mutations that lead to new functions do so by adversely affecting ancestral functionality. To the extent that purifying selection maintains the ancestral function in both duplicated genes, the same purifying selection would act against the evolution of new functionality in either of the duplicates.

Taken together our results shed light onto the lifecycle of a gene in eukaryotic genomes. The common view of the long-term evolution of duplicate genes is that they are redundant immediately upon duplication, quickly undergo subfunctionalization and some neofunctionalization, and over long time periods begin to behave as singleton genes. This notion is attractive as it provides for a steady state description of gene fate in the genome. However, here we show that, when tested in rich medium, duplicate gene pairs maintain substantial redundancy, acquire little new functionality, and do not behave as singleton genes even after ∼100 million years of evolution.

## Materials and Methods

### Obtaining yeast ORFs

We obtained a list of all yeast ORFs from SGD [25] on 11/14/2006. We retained the 5781 ORFs listed as ‘Verified’ and ‘Uncharacterized’. We excluded ‘Dubious’ ORFs as these are highly unlikely to be protein coding and ‘Verified∥silenced_gene’ as these four are associated with the mating type cassette.

### Strain Building Overview

We began with a diploid strain in which gene A was deleted and replaced with a drug marker, and a second diploid strain in which gene B was deleted and replaced with a different drug marker. We sporulated these strains, crossed them, and selected diploid strains that contained both drug markers and are therefore contain heterozygous deletions of both genes. This diploid heterozygous strain was sporulated and the meiotic products were separated by tetrad dissection. The ploidy of these strains was confirmed by polymerase chain reaction (PCR), and the genotype was confirmed by observing growth on various drug-containing media. The yeast strains were grown on solid or in liquid standard rich medium (YPD) [49],[50] at 30°C unless otherwise stated.

### Strain Building Details

Individual diploid heterozygous deletion strains were obtained either from the existing collection of yeast deletion strains [51] or were built using the following protocol. For cases in which we built the individual deletion strains, we began with the S288c-derived strain S1001 (*MATa/MATα gal2/gal2*). The deletion cassette includes either the geneticin resistance drug marker *kanMX* of the plasmid pUG6 [52] or the nourseothricin resistance drug marker *natMX* of the plasmid pAG36 [53]. Flanking the drug resistance marker are 100 base pair regions of DNA necessary to guide the homologous recombination and delete the specific gene of interest.

For each gene to be deleted, we designed a pair of 120 base primers. These primers contain 100 bases homologous to the region immediately upstream of the start codon or downstream of the stop codon of the gene to be deleted, along with 20 bases homologous to the region of the plasmid flanking the drug marker (5′-CCTTGACAGTCTTGACGTGC-3′ for the upstream primer and 5′-CGCACTTAACTTCGCATCTG-3′ for the downstream primer). In using these pairs of primers to amplify the drug resistance marker from the plasmid by means of PCR, we created DNA fragments capable of selectively deleting each of our genes of interest. We transformed the S1001 strain with the product of the PCR and selected for transformants on medium supplemented with geneticin (400 µg/ml) (Invitrogen, Carlsbad, California, United States) or nourseothricin (100 µg/ml) (Werner BioAgents, Jena, Germany).

Individual deletion strains obtained from the deletion collection all contain a geneticin resistance cassette and so, for each pair of genes investigated, one of the drug markers was switched from *kanMX* to *natMX*. The drug markers are flanked by regions common to both markers and thus are amenable to swapping by means of amplification, transformation, and homologous recombination. This was achieved by using PCR to amplify the nourseothricin resistance marker using the 20 base primers listed above, transforming the strains with this PCR product, and selecting for transformants on medium containing nourseothricin.

The two strains containing deletions of genes of a specific pair were brought together by sporulation and crossing. The strains were sporulated by taking cells grown overnight in liquid YPD and putting them into 0.5% potassium acetate for 5 days. The resulting cell mixtures, each of which contain some haploid cells, were brought together and grown in liquid YPD overnight to allow the haploid cells to mate. They were then struck out on YPD with geneticin (400 µg/ml) and nourseothricin (100 µg/ml) to select for the diploid heterozygous double deletion strain.

The presence of the correct gene deletions in the diploid heterozygous double deletion strain were confirmed using PCR. We designed [54] and synthesized a pair of primers to confirm both the up and the down recombination junction. Each pair consisted of an outside primer unique to the upstream or downstream region surrounding a gene and an inside primer within the *kanMX* or *natMX* marker. These primer pairs were only able to yield a product from PCR if, in fact, the correct gene had been deleted. These outside primers were designed using primer3 [55] to regions ∼400–600 bases upstream and downstream of the start and stop codons and were paired with the inside primers for the *kanMX* (5′-GCCTCGAAACGTGAGTCTTT-3′ for the upstream junction and 5′-TTGCAGTTTCATTTGATGCTC-3′ for the downstream junction) or *natMX* (5′-AGCCGTGTCGTCAAGAGTG-3′ for the upstream junction and 5′-GAGCAGGCGCTCTACATGA-3′ for the downstream junction) cassette.

These diploid heterozygous double deletion strains were sporulated as previously described, and six tetrads for each strain were dissected yielding 24 colonies. Strains derived from these 24 colonies were confirmed to be haploid by PCR [56], and the genotype was determined by monitoring growth on YPD, YPD+geneticin, YPD+nourseothricin, and YPD+geneticin+nourseothricin to be either a wild type strain, a strain with an individual deletion in gene A, a strain with an individual deletion in gene B, or a strain with a deletion in gene A and gene B.

### Measuring Fitness

The growth rate of these strains in liquid YPD was determined by monitoring cell density as has been previously described [27],[57]. The strains were grown overnight in YPD and then diluted to an OD_600_ of 0.01 or lower in a final volume of 100 µl using a Biomek FX Laboratory Automation Workstation (Beckman Coulter, Allendale, New Jersey, United States) and in 96-well plates (Nunc, Rochester, New York, United States). The Tecan GENios microplate reader (Tecan, Mannedorf/Zurich, Switzerland) maintained the cultures at constant temperature and shaking, and took optical density readings every 15 minutes. The growth rate, or doubling time during the period of exponential growth, was determined by fitting an exponential curve using a custom built software package [27],[57].

The growth rates for strains carrying gene deletions of genes in the proxy set were determined in much the same fashion. Of the 305 genes in the proxy set, 67 are essential, 229 were obtained from the collection of homozygous diploid deletion strains, 5 were obtained from the collection of haploid MAT alpha deletion strains, and 4 were not found in any of the collections. For each strain we measured the growth rate at least three times and averaged across the replicates.

The bulk of the strains carrying single gene deletions of singleton genes grows at the same rate as the wild type strain. We used the ksdensity function in MATLAB (The MathWorks Inc., Natick, Massachusetts, United States) and fitness data for strains carrying single gene deletions [26] to build a density distribution of the fitness values for strains carrying deletions of singleton genes. The peak value in this distribution is highly similar between the distribution for all singleton genes and the distribution for our proxy set (1.015 and 1.0087 respectively). This suggests that the bulk of the strains carrying single gene deletions of the genes in our proxy set grow like the wild type strain. In the same fashion, we then estimated the density across our growth rate data for strains carrying single gene deletions of the genes in our proxy set. We determined the growth rate with the highest density and used that as an estimate of the growth rate of the wild type strain. We then calculated the fitness of each deletion strain relative to the wild type strain. This process was carried out separately for the haploid and diploid deletion strains as it is known the haploid and diploid wild type strains grow at different rates.

### Accounting for Duplicate Pairs in which a Gene Is Essential

Some duplicate gene pairs contain an essential gene. For these pairs we assume the double gene deletion strain would also be lethal. Of the 257 pairs in which neither gene was essential, we have data for only 201 of the pairs. We wanted to include the correct proportion of these pairs in our distribution of fitness values for strains carrying double gene deletions. Separately, we determined the amount to be included for the subset of ribosomal gene pairs ((36/42)*4≈3) and non-ribosomal gene pairs ((165/215)*28≈21).

## Supporting Information

Figure S1 Comparing single gene deletion fitness values for singleton and duplicate gene pairs. We graphed the maximum and minimum fitness values for strains carrying single gene deletions of the genes within each of the 90 singleton gene pairs (red) and 257 duplicate gene pairs (blue) in which neither gene is essential.
(1.06 MB EPS)

Figure S2 Testing for evidence of gained functionality among subsets of the duplicate gene data. The empirical cumulative distributions display the fitness values for strains carrying single gene deletions of non-ribosomal genes within the proxy set and for strains carrying double gene deletions of duplicate genes. The figures show distributions of fitness values for (A) non-ribosomal genes from the proxy set and non-ribosomal duplicate gene pairs, (B) non-ribosomal genes from the proxy set and non-ribosomal duplicate gene pairs derived from the WGD event, and (C) non-ribosomal genes from the proxy set and non-ribosomal duplicate gene pairs derived from SSD events. We also include the empirical cumulative distribution of the expected fitness values for double deletions of paired singleton genes (grey).
(2.56 MB EPS)

Figure S3 Examining alternatives to our proxy set for ancestral progenitors. These are cumulative distribution functions of the fitness values for strains carrying single gene deletions of genes in our proxy set (red), as well as three alternative sets. The singleton genes in our proxy set (i) show evidence of a duplication event in one of 12 related yeast species and (ii) do not show evidence of a *S. cerevisiae* lineage specific gene loss. The first alternative proxy set (grey) contains all singleton genes, the second alternative proxy set (green) eliminates the second criterion, and the third alternative proxy set (yellow) adds a requirement that orthologs of the genes in the set must be present in all 12 related species. We used fitness values from Steinmetz et al. balanced to include the ratio of essential and non-essential genes observed within our set of duplicate gene pairs. The lists of essential and non-essential genes were communicated by those at the *Saccharomyces* Genome Deletion Project and provide the best estimate of the proportion of genes that are essential in rich medium (23.6% of all singleton genes, 75.0% of ribosomal and 23.4% of non-ribosomal singleton genes). The Steinmetz et al. data set does not have fitness data for every deletion of a non-essential singleton gene (e.g. for the complete set of singleton genes they have data for 3 of 3 non-essential ribosomal singleton genes and 1724 of 1896 non-essential non-ribosomal singleton genes). To maintain the correct ratio of essential to non-essential singleton genes within the data set, only the appropriate proportion of the essential singleton genes (*W* = 0) were included in the distribution (e.g. for the complete set of singletons genes we include for the ribosomal subset (9 * 3/3 = 9), for the non-ribosomal subset (579 * 1724/1896 = ∼526)).
(1.38 MB EPS)

Dataset S1 Duplicate gene pairs.
(0.18 MB PDF)

Dataset S2 Paired singleton genes.
(0.11 MB PDF)

Dataset S3 Genes in proxy set.
(0.08 MB PDF)
