# Supplementary material for: Pervasive and Persistent Redundancy among Duplicated Genes in Yeast
Source: PLoS Genet. 2008 Jul 4;4(7):e1000113. doi: 10.1371/journal.pgen.1000113 (PMC2440806; doi:10.1371/journal.pgen.1000113)
Supplement: Dataset S1 — Duplicate gene pairs. (0.18 MB PDF) [file pgen.1000113.s004.pdf]

ORF1	ORF2	ORF1_fit	ORF2_fit	ORF1ORF2_fit
YMR205C	YGR240C	0.36	0.86	0
YHR183W	YGR256W	0.39	0.97	0
YPL249C-A	YMR194W	0.46	0.78	0
YKL006W	YHL001W	0.46	0.90	0
YGR162W	YGL049C	0.46	1.00	0
YER117W	YBL087C	0.52	0.79	0
YGL189C	YER131W	0.53	0.90	0
YPR043W	YJR094W-A	0.53	0.87	0
YOR293W	YMR230W	0.54	0.75	0
YOR096W	YNL096C	0.56	0.90	0
YOL115W	YNL299W	0.59	1.00	0
YML024W	YDR447C	0.61	0.83	0
YML063W	YLR441C	0.65	0.87	0
YJR145C	YHR203C	0.66	0.76	0
YPL090C	YBR181C	0.67	0.73	0
YPR159W	YGR143W	0.67	1.00	0
YLR185W	YDR500C	0.68	0.81	0
YML073C	YLR448W	0.69	0.77	0
YNL162W	YHR141C	0.70	0.62	0
YBR084C-A	YBL027W	0.73	0.55	0
YOR182C	YLR287C-A	0.75	0.83	0
YOL121C	YNL302C	0.76	0.64	0
YKR057W	YJL136C	0.78	0.61	0
YML026C	YDR450W	0.79	0.71	0
YLR388W	YDL061C	0.80	0.74	0
YHR066W	YDR312W	0.84	1.00	0
YER102W	YBL072C	0.85	0.64	0
YIL069C	YER074W	0.88	0.44	0
YNL055C	YIL114C	0.89	1.01	0
YOR167C	YLR264W	0.90	0.77	0
YLR367W	YJL190C	0.90	0.69	0
YMR238W	YKL046C	0.94	0.99	0
YKL008C	YHL003C	0.95	0.95	0
YPL220W	YGL135W	0.95	0.53	0
YIL138C	YNL079C	0.95	0.41	0
YLR180W	YDR502C	0.96	1.03	0
YKL156W	YHR021C	0.96	0.95	0
YJR103W	YBL039C	0.97	0.81	0
YPL240C	YMR186W	0.98	1.00	0
YJL191W	YCR031C	0.98	0.88	0
YER105C	YBL079W	0.99	0.50	0
YLR328W	YGR010W	0.99	1.00	0
YNL030W	YBR009C	1.00	0.98	0
YKR067W	YBL011W	1.00	0.96	0
YER177W	YDR099W	1.01	1.02	0
YIL066C	YER070W	1.01	0.55	0

YDR012W	YBR031W	1.01	0.81	0
YJR148W	YHR208W	1.02	0.89	0
YPL135W	YOR226C	1.02	1.02	0
YHR033W	YDR300C	0.96	0.44	0.22
YOR295W	YMR233W	0.29	1.01	0.26
YEL054C	YDR418W	0.69	0.53	0.26
YNR027W	YEL029C	1.01	0.24	0.30
YEL042W	YER005W	0.97	1.00	0.43
YCL057W	YKL134C	1.05	0.53	0.46
YPR032W	YBL106C	0.95	0.99	0.53
YKR018C	YJL082W	1.00	0.99	0.54
YIR021W	YKL011C	0.71	0.98	0.57
YLR061W	YFL034C-A	0.66	1.00	0.63
YML061C	YHR031C	0.94	0.97	0.64
YOR270C	YMR054W	0.91	0.98	0.67
YPL152W	YIL153W	0.94	0.96	0.69
YIL052C	YER056C-A	0.94	0.72	0.69
YKR050W	YJL129C	0.94	0.96	0.72
YPL189W	YGL084C	0.95	0.76	0.74
YHR010W	YDR471W	0.95	0.87	0.76
YLR350W	YGR038W	1.01	1.00	0.77
YLR333C	YGR027C	0.97	0.92	0.78
YGR155W	YGR012W	0.76	0.93	0.78
YGL054C	YBR210W	0.96	1.01	0.80
YLR130C	YGL255W	0.93	0.96	0.81
YNL197C	YDL224C	0.96	1.01	0.83
YHR097C	YDR348C	0.99	0.91	0.85
YCL051W	YDR528W	0.91	0.99	0.87
YPL037C	YDR252W	0.93	0.96	0.87
YKL087C	YAL039C	0.94	0.88	0.87
YMR153W	YDL088C	0.94	0.93	0.87
YJR048W	YEL039C	0.95	0.99	0.88
YBR037C	YBR024W	0.89	1.01	0.89
YMR120C	YLR028C	1.03	1.08	0.90
YIL135C	YNL074C	0.97	0.96	0.90
YNL130C	YHR123W	1.00	1.00	0.91
YJR133W	YDR399W	0.95	0.93	0.91
YHR108W	YDR358W	0.98	1.03	0.91
YLR406C	YDL075W	0.93	1.01	0.91
YLR324W	YGR004W	0.92	0.98	0.91
YER139C	YDR066C	0.86	1.00	0.91
YLR344W	YGR034W	0.95	0.94	0.92
YML022W	YDR441C	0.92	0.93	0.92
YHR061C	YDR309C	1.02	1.00	0.92
YJL137C	YKR058W	0.96	0.94	0.92
YGL133W	YPL216W	0.92	0.99	0.92
YDR035W	YBR249C	0.98	0.97	0.93

YLR177W	YDR505C	1.00	0.97	0.93
YGR066C	YBR105C	0.97	0.96	0.94
YOR338W	YAL034C	0.96	0.96	0.94
YNL117W	YIR031C	0.94	0.99	0.94
YHR204W	YJR131W	1.00	0.98	0.94
YPR069C	YLR146C	0.98	0.99	0.94
YEL017C-A	YCR024C-A	1.00	0.92	0.94
YPL060W	YOR334W	0.94	0.95	0.95
YNL257C	YHR155W	0.97	0.99	0.95
YDL204W	YDR233C	1.00	0.99	0.95
YMR310C	YGR283C	0.97	0.99	0.95
YGL222C	YER035W	0.94	0.97	0.95
YHR103W	YDR351W	1.00	0.97	0.95
YIL056W	YER064C	0.98	0.98	0.95
YOR173W	YLR270W	0.96	0.97	0.95
YGR184C	YLR024C	1.00	0.95	0.95
YDR001C	YBR001C	0.98	0.99	0.95
YGL019W	YOR039W	0.97	0.92	0.96
YPR074C	YBR117C	0.96	1.00	0.96
YKR053C	YJL134W	1.04	0.99	0.96
YLR183C	YDR501W	0.97	0.98	0.96
YJL181W	YJR030C	0.97	0.97	0.96
YJL058C	YBR270C	1.00	0.99	0.96
YDR214W	YNL281W	0.97	0.96	0.96
YPL023C	YGL125W	1.00	1.00	0.96
YER145C	YBR207W	0.93	0.90	0.96
YKL050C	YMR031C	1.00	1.01	0.97
YPR194C	YJL212C	1.00	0.99	0.97
YDR206W	YLR233C	1.01	1.01	0.97
YGR035C	YLR346C	0.97	0.94	0.97
YOL043C	YAL015C	0.98	0.97	0.97
YKL039W	YHL017W	0.99	1.00	0.97
YGR204W	YBR084W	0.95	1.00	0.97
YDL181W	YDL130W-A	1.08	1.02	0.97
YDR003W	YBR005W	1.01	0.99	0.97
YDR530C	YCL050C	0.97	0.97	0.97
YJR015W	YGR197C	0.98	0.98	0.97
YOR180C	YLR284C	0.95	0.96	0.98
YJR054W	YML047C	1.01	1.02	0.98
YMR206W	YNR014W	0.98	0.98	0.98
YPR172W	YLR456W	0.97	0.97	0.98
YFR039C	YGL228W	1.00	0.97	0.98
YMR115W	YKL133C	1.01	1.01	0.98
YOR312C	YMR242C	0.99	0.99	0.98
YOR307C	YJL193W	0.97	0.99	0.98
YLR133W	YDR147W	1.03	1.02	0.98
YMR053C	YKL072W	0.98	0.98	0.98

YPL187W	YGL089C	0.99	0.97	0.98
YDR018C	YBR042C	1.00	1.00	0.98
YPL191C	YGL082W	0.99	1.01	0.98
YPL212C	YGL063W	0.98	1.00	0.98
YHL039W	YPL208W	1.00	1.00	0.98
YOR040W	YDR272W	1.00	0.99	0.98
YML070W	YFL053W	0.99	0.99	0.98
YDR514C	YCL036W	0.98	0.99	0.98
YKL103C	YHR113W	1.01	1.02	0.98
YMR264W	YML101C	0.98	0.98	0.98
YOL017W	YFR013W	0.97	1.03	0.98
YBL059W	YER093C-A	1.04	1.02	0.99
YGR231C	YGR132C	1.00	0.99	0.99
YLR354C	YGR043C	0.98	0.99	0.99
YPL105C	YBR172C	0.93	0.97	0.99
YDR273W	YOR042W	1.04	1.01	0.99
YLR454W	YPR117W	1.01	1.00	0.99
YOR047C	YDR277C	0.98	1.02	0.99
YPL004C	YGR086C	0.99	0.96	0.99
YDR009W	YBR020W	1.01	1.01	0.99
YOR375C	YAL062W	0.98	0.98	0.99
YNL145W	YDR461W	0.98	0.99	0.99
YLR361C	YOR137C	1.02	0.99	0.99
YDR503C	YDR284C	0.99	0.99	0.99
YGR088W	YDR256C	1.01	0.98	0.99
YMR295C	YGR273C	0.98	0.99	0.99
YDR458C	YML034W	1.00	0.97	0.99
YJR116W	YPR114W	0.98	1.00	0.99
YKL093W	YMR081C	1.01	1.03	0.99
YGR142W	YPR158W	1.01	1.01	0.99
YAL028W	YOR324C	1.00	0.98	0.99
YLR136C	YDR151C	0.98	1.02	0.99
YEL016C	YCR026C	0.98	0.98	0.99
YBR197C	YPL077C	1.00	0.99	0.99
YOR171C	YLR260W	0.98	0.99	0.99
YIL057C	YER067W	0.93	0.95	1.00
YPR006C	YER065C	1.03	1.02	1.00
YLR080W	YFL048C	0.99	0.98	1.00
YGL162W	YPR009W	0.99	0.98	1.00
YGL060W	YBR216C	0.99	0.98	1.00
YLR058C	YBR263W	1.02	1.01	1.00
YBR273C	YJL048C	0.97	0.98	1.00
YOR377W	YGR177C	0.98	1.01	1.00
YML075C	YLR450W	1.01	1.03	1.00
YMR271C	YML106W	1.00	1.02	1.01
YLR258W	YFR015C	0.98	1.01	1.01
YNR019W	YCR048W	1.00	1.01	1.01

YNL156C	YHR133C	0.98	1.02	1.01
YPR157W	YGR141W	1.01	0.98	1.01
YPR193C	YEL066W	0.97	1.01	1.01
YGL056C	YBR214W	1.02	1.02	1.01
YDR109C	YNL249C	1.01	1.02	1.01
YPL250C	YMR195W	1.03	1.04	1.01
YAL037W	YOR342C	1.00	0.98	1.01
YPL130W	YOR214C	0.98	0.93	1.02
YLR108C	YDR132C	1.01	1.02	1.02
YLR327C	YGR008C	1.04	1.03	1.02
YPL087W	YBR183W	1.07	1.00	1.02
YOR219C	YHR028C	1.01	1.04	1.03
YOR371C	YAL056W	1.03	1.03	1.04
YGR230W	YHR152W	1.00	1.03	1.07
YMR118C	YKL141W			
YKL180W	YJL177W			
YJL026W	YGR180C			
YDR224C	YBL002W			
YOR110W	YNL108C			
YPL028W	YIL160C			
YJL174W	YDL049C			
YML065W	YLR442C			
YMR267W	YBR011C			
YOR347C	YAL038W			
YMR101C	YBR002C			
YPR081C	YBR121C			
YIL003W	YGL091C			
YJL031C	YKL019W			
YHR088W	YNL075W			
YCR052W	YNR023W			
YPL143W	YOR234C			
YOL120C	YNL301C			
YIL106W	YFL034C-B			
YOR048C	YGL173C			
YDL164C	YOR005C			
YGR029W	YPR037C			
YER171W	YPL008W			
YLR008C	YNL328C			
YLR457C	YPR174C			
YHR091C	YDR341C			
YJR047C	YEL034W			
YMR121C	YLR029C			
YHR011W	YDR023W			
YDR072C	YKL004W			
YDL104C	YKR038C			
YMR180C	YPL228W			
YMR142C	YDL082W			

YDL191W	YDL136W
YPR102C	YGR085C
YHR132W-A	YNL157W
YMR119W	YNL008C
YGR071C	YLR373C
YGR049W	YLR356W
YBL091C-A	YER120W
YHR146W	YNL173C
YJL083W	YKR019C
YDR379W	YOR127W
YBL101C	YPR030W
YIR033W	YKL020C
YDR515W	YCL037C
YDL175C	YIL079C
YLR371W	YGR070W
YJR005C-A	YGR169C-A
YPL256C	YMR199W
YBL009W	YGL021W
YPL079W	YBR191W
YGL224C	YER037W
YDR025W	YBR048W
YOL059W	YDL022W
YPL171C	YHR179W
YHR005C	YER020W
YHR199C	YHR198C
YCL026C-B	YCL026C-A
YNL335W	YFL061W
YOR387C	YGL258W
YJL038C	YJL037W
YPL279C	YOR390W
YGL012W	YNL280C
YOL027C	YPR125W
YOL082W	YOL083W
YER087C-B	YER019C-A
YHR032W	YDR338C
YHR213W-B	YAR064W
YOR072W-B	YOL013W-A
YOR147W	YHR194W
YIL038C	YPR072W
YER188C-A	YOL166W-A
YPL184C	YML117W
YLR027C	YKL106W
YLR308W	YLR307W
YOR286W	YOR285W
YBL043W	YJR115W
YPL112C	YOR193W
YMR181C	YPL229W

YGL071W	YPL202C
YIL158W	YKR100C
YDL184C	YDL133C-A
YIR018W	YOL028C
YKR091W	YOR083W
YBR278W	YJL065C
YHR160C	YGR239C
YDR259C	YOR028C
