# Supplementary material for: Pervasive and Persistent Redundancy among Duplicated Genes in Yeast
Source: PLoS Genet. 2008 Jul 4;4(7):e1000113. doi: 10.1371/journal.pgen.1000113 (PMC2440806; doi:10.1371/journal.pgen.1000113)
Supplement: Dataset S2 — Paired singleton genes. (0.11 MB PDF) [file pgen.1000113.s005.pdf]

ORF1	ORF2	ORF1_fit	ORF2_fit	ORF1ORF2_fit
YPL097W	YPL161C	0.47	0.81	0.43
YPR057W	YBR200W	0.77	0.50	0.49
YPL148C	YPR023C	0.58	0.99	0.53
YIL162W	YNL139C	0.99	0.56	0.54
YER044C	YOL147C	0.64	0.94	0.54
YCR020C-A	YGL246C	1.03	0.60	0.59
YML062C	YFR036W	0.78	0.90	0.60
YDR392W	YIL040W	0.84	0.80	0.62
YER141W	YOR035C	0.78	0.74	0.65
YNL199C	YJR043C	0.76	0.95	0.72
YKL119C	YBR076W	0.69	1.00	0.72
YHR041C	YJR053W	0.72	0.96	0.72
YBR035C	YKL110C	0.96	0.78	0.74
YLR239C	YDR176W	0.71	0.80	0.77
YGL066W	YAL014C	0.76	1.00	0.79
YAR015W	YMR039C	0.97	0.88	0.82
YNL294C	YEL003W	0.93	0.99	0.83
YNL230C	YJR117W	0.94	0.93	0.85
YGR063C	YLL042C	0.86	0.99	0.87
YHR134W	YLR421C	0.89	0.96	0.88
YOR017W	YHR193C	1.00	0.91	0.88
YDR403W	YGR229C	0.99	0.91	0.90
YDR456W	YJL209W	1.00	0.87	0.90
YGR166W	YFL007W	0.93	0.97	0.90
YDR354W	YKL185W	0.92	0.98	0.90
YDR315C	YLR368W	1.01	0.94	0.90
YEL053C	YBR120C	1.02	0.92	0.90
YMR234W	YNL070W	1.00	0.95	0.91
YER046W	YEL062W	0.99	0.94	0.91
YOR243C	YBR258C	0.94	1.00	0.92
YDR120C	YLR176C	0.98	0.99	0.92
YFL013C	YBR188C	0.98	0.99	0.94
YKR097W	YPL003W	1.00	1.00	0.94
YAL055W	YGR144W	0.95	1.01	0.95
YML006C	YML095C	0.96	0.96	0.95
YLL060C	YPR020W	1.00	0.95	0.95
YIL076W	YLR267W	0.94	0.96	0.95
YCR075C	YJL201W	0.95	0.98	0.95
YHR184W	YHL016C	0.97	0.99	0.96
YGR236C	YPL046C	0.95	1.02	0.96
YBR045C	YJR082C	1.01	1.00	0.96
YBR201W	YEL052W	0.97	0.97	0.96
YDR007W	YHL006C	0.97	1.02	0.96
YIR032C	YAL031C	0.99	0.99	0.96
YOR274W	YPL127C	0.98	0.99	0.96
YJR069C	YDL122W	0.98	0.97	0.97

YNL159C	YDR143C	0.97	0.99	0.97
YIL020C	YMR302C	0.99	0.98	0.97
YMR274C	YNL292W	0.98	0.98	0.97
YHR109W	YIL065C	0.99	0.98	0.97
YER115C	YLR390W	0.98	1.01	0.97
YDR158W	YBR290W	0.99	1.00	0.97
YGR284C	YLR363C	0.99	1.00	0.97
YDR110W	YHR185C	0.98	0.99	0.97
YDL231C	YPL192C	1.00	1.00	0.97
YOR265W	YLR094C	0.99	0.99	0.98
YGL124C	YER106W	0.97	1.01	0.98
YDR058C	YCL055W	1.00	0.98	0.98
YGR194C	YMR020W	0.98	0.98	0.98
YDL076C	YDR281C	0.96	1.01	0.98
YLR250W	YGL067W	0.97	0.98	0.98
YOL049W	YOR087W	0.97	1.00	0.98
YJL071W	YJR092W	0.99	1.00	0.98
YOR253W	YKL067W	0.99	0.99	0.98
YDR057W	YHL022C	1.00	1.00	0.98
YJR010C-A	YGL175C	1.00	0.96	0.98
YDL135C	YGR196C	0.98	0.99	0.98
YGL090W	YKL130C	0.99	1.01	0.98
YIR017C	YBR272C	0.99	0.99	0.98
YER149C	YGL121C	0.99	1.00	0.98
YAL002W	YDR481C	0.93	1.01	0.98
YMR044W	YJR094C	0.98	0.97	0.99
YBR228W	YLR135W	0.96	1.00	0.99
YER180C	YOR132W	0.99	0.97	0.99
YDL077C	YLR151C	0.96	0.99	0.99
YJR135C	YOR184W	0.98	1.01	0.99
YGR217W	YLR292C	1.01	1.00	0.99
YPR073C	YGL017W	0.98	1.00	0.99
YGR244C	YGR044C	0.99	1.00	0.99
YOR126C	YDR073W	1.01	1.02	0.99
YFL027C	YBR176W	0.99	1.01	0.99
YIL132C	YPR167C	0.98	0.98	0.99
YJL146W	YOR202W	1.01	0.99	1.00
YHL030W	YCR020C	0.99	1.03	1.00
YDR179C	YBR083W	0.99	1.04	1.01
YMR065W	YKL184W	0.99	0.98	1.01
YIL037C	YKL025C	0.95	1.02	1.01
YPL111W	YLR084C	1.03	1.02	1.02
YKR056W	YJL100W	1.01	1.01	1.02
YMR036C	YNL212W	1.03	1.04	1.08
