# Supplementary material for: Pervasive and Persistent Redundancy among Duplicated Genes in Yeast
Source: PLoS Genet. 2008 Jul 4;4(7):e1000113. doi: 10.1371/journal.pgen.1000113 (PMC2440806; doi:10.1371/journal.pgen.1000113)
Supplement: Dataset S3 — Genes in proxy set. (0.08 MB PDF) [file pgen.1000113.s006.pdf]

| Genes in proxy set | |
|---|---|
| YGL103W | 0 |
| YGL030W | 0 |
| YPL131W | 0 |
| YAL001C | 0 |
| YBL020W | 0 |
| YBL097W | 0 |
| YBR079C | 0 |
| YBR102C | 0 |
| YBR110W | 0 |
| YBR152W | 0 |
| YBR236C | 0 |
| YDL147W | 0 |
| YDR081C | 0 |
| YDR166C | 0 |
| YDR180W | 0 |
| YDR182W | 0 |
| YDR246W | 0 |
| YDR376W | 0 |
| YDR381W | 0 |
| YDR407C | 0 |
| YDR434W | 0 |
| YDR478W | 0 |
| YDR499W | 0 |
| YDR531W | 0 |
| YEL055C | 0 |
| YEL058W | 0 |
| YER022W | 0 |
| YER126C | 0 |
| YER148W | 0 |
| YER168C | 0 |
| YGL065C | 0 |
| YGL097W | 0 |
| YGL233W | 0 |
| YGR002C | 0 |
| YGR140W | 0 |
| YGR186W | 0 |
| YGR245C | 0 |
| YHR170W | 0 |
| YHR188C | 0 |
| YIL129C | 0 |
| YJL087C | 0 |
| YJR041C | 0 |
| YKR068C | 0 |
| YKR079C | 0 |
| YLR071C | 0 |
| YLR103C | 0 |

| | |
|---|---|
| YLR215C | 0 |
| YLR272C | 0 |
| YML049C | 0 |
| YML091C | 0 |
| YMR200W | 0 |
| YMR227C | 0 |
| YMR270C | 0 |
| YNL026W | 0 |
| YNL126W | 0 |
| YNL132W | 0 |
| YNL152W | 0 |
| YNL172W | 0 |
| YNL244C | 0 |
| YNL258C | 0 |
| YOL139C | 0 |
| YOR149C | 0 |
| YOR206W | 0 |
| YPL012W | 0 |
| YPL117C | 0 |
| YPL231W | 0 |
| YPR085C | 0 |
| YNL138W | 0.35 |
| YPL118W | 0.42 |
| YLR312W-A | 0.42 |
| YKR082W | 0.43 |
| YDR027C | 0.52 |
| YBR026C | 0.52 |
| YBR081C | 0.56 |
| YNL054W | 0.56 |
| YBL058W | 0.57 |
| YBR039W | 0.59 |
| YDL013W | 0.64 |
| YER116C | 0.64 |
| YDL115C | 0.64 |
| YDR320C | 0.65 |
| YFL025C | 0.65 |
| YOR073W | 0.67 |
| YIL033C | 0.67 |
| YLL006W | 0.70 |
| YML128C | 0.71 |
| YER154W | 0.74 |
| YDR159W | 0.74 |
| YLR384C | 0.74 |
| YMR032W | 0.74 |
| YDL117W | 0.79 |
| YCR081W | 0.79 |
| YCR044C | 0.79 |

| | |
|---|---|
| YDR336W | 0.80 |
| YNL241C | 0.80 |
| YEL009C | 0.81 |
| YNL236W | 0.81 |
| YGL234W | 0.81 |
| YJR074W | 0.82 |
| YDR414C | 0.84 |
| YHR134W | 0.84 |
| YLR148W | 0.85 |
| YNL190W | 0.85 |
| YGL066W | 0.86 |
| YDR080W | 0.86 |
| YDR316W | 0.86 |
| YML097C | 0.87 |
| YNL239W | 0.87 |
| YJL204C | 0.87 |
| YMR216C | 0.87 |
| YBR255W | 0.88 |
| YOR322C | 0.88 |
| YCR017C | 0.88 |
| YOL064C | 0.88 |
| YGR229C | 0.88 |
| YBR229C | 0.89 |
| YPR147C | 0.89 |
| YGL027C | 0.89 |
| YCL045C | 0.89 |
| YDL077C | 0.89 |
| YBR235W | 0.89 |
| YPL054W | 0.89 |
| YGL244W | 0.89 |
| YOR258W | 0.89 |
| YDL089W | 0.90 |
| YBL060W | 0.90 |
| YDR400W | 0.90 |
| YKL184W | 0.90 |
| YLR392C | 0.90 |
| YDR408C | 0.90 |
| YDL110C | 0.90 |
| YKR056W | 0.90 |
| YOR155C | 0.91 |
| YPL022W | 0.91 |
| YDR330W | 0.91 |
| YGL211W | 0.91 |
| YKL207W | 0.91 |
| YOR274W | 0.91 |
| YDR031W | 0.92 |
| YNL168C | 0.92 |

| | |
|---|---|
| YDL219W | 0.92 |
| YNL294C | 0.92 |
| YDL057W | 0.92 |
| YBR131W | 0.92 |
| YDL046W | 0.92 |
| YER042W | 0.92 |
| YOR124C | 0.92 |
| YDR329C | 0.92 |
| YDR428C | 0.92 |
| YMR231W | 0.92 |
| YFL034W | 0.92 |
| YDR411C | 0.93 |
| YKR044W | 0.93 |
| YOR126C | 0.93 |
| YFL030W | 0.93 |
| YEL015W | 0.93 |
| YOR349W | 0.93 |
| YFR047C | 0.93 |
| YFL047W | 0.93 |
| YGL124C | 0.93 |
| YEL023C | 0.93 |
| YBL102W | 0.93 |
| YMR202W | 0.94 |
| YML038C | 0.94 |
| YEL001C | 0.94 |
| YOR132W | 0.94 |
| YNL273W | 0.94 |
| YML050W | 0.94 |
| YLL040C | 0.95 |
| YDR279W | 0.95 |
| YGL037C | 0.95 |
| YBR007C | 0.95 |
| YML059C | 0.95 |
| YDR108W | 0.95 |
| YHR189W | 0.95 |
| YPR097W | 0.95 |
| YML055W | 0.96 |
| YLR138W | 0.96 |
| YDR481C | 0.96 |
| YDL171C | 0.96 |
| YDR071C | 0.96 |
| YKL094W | 0.96 |
| YIL145C | 0.96 |
| YMR137C | 0.96 |
| YLR271W | 0.97 |
| YIL077C | 0.97 |
| YKL217W | 0.97 |

| | |
|---|---|
| YHR140W | 0.97 |
| YJL123C | 0.97 |
| YKR093W | 0.97 |
| YLR172C | 0.97 |
| YIL047C | 0.97 |
| YLR381W | 0.97 |
| YNL297C | 0.97 |
| YLR097C | 0.97 |
| YHR029C | 0.97 |
| YBR115C | 0.97 |
| YIL049W | 0.97 |
| YPL120W | 0.97 |
| YKR069W | 0.97 |
| YGR235C | 0.97 |
| YLR422W | 0.98 |
| YJR099W | 0.98 |
| YNL275W | 0.98 |
| YMR027W | 0.98 |
| YEL052W | 0.98 |
| YLL057C | 0.98 |
| YKL084W | 0.98 |
| YHR009C | 0.98 |
| YBL066C | 0.98 |
| YOL096C | 0.98 |
| YIL007C | 0.98 |
| YIR007W | 0.98 |
| YNL311C | 0.98 |
| YJL093C | 0.98 |
| YLR455W | 0.98 |
| YMR012W | 0.99 |
| YIL162W | 0.99 |
| YMR259C | 0.99 |
| YPR045C | 0.99 |
| YNR074C | 0.99 |
| YCL030C | 0.99 |
| YHR008C | 0.99 |
| YNR018W | 0.99 |
| YNL127W | 0.99 |
| YLR142W | 0.99 |
| YMR247C | 0.99 |
| YPL246C | 0.99 |
| YPR089W | 0.99 |
| YPR091C | 0.99 |
| YNL253W | 0.99 |
| YMR130W | 0.99 |
| YIL016W | 1.00 |
| YIR023W | 1.00 |

| | |
|---|---|
| YFL014W | 1.00 |
| YLR299W | 1.00 |
| YDL236W | 1.00 |
| YNL123W | 1.00 |
| YOL008W | 1.00 |
| YHR202W | 1.00 |
| YNL129W | 1.00 |
| YOL159C | 1.00 |
| YIR017C | 1.00 |
| YER052C | 1.00 |
| YFR025C | 1.00 |
| YBR179C | 1.00 |
| YGL138C | 1.00 |
| YKL215C | 1.00 |
| YJR100C | 1.00 |
| YJR040W | 1.00 |
| YJR154W | 1.00 |
| YBR187W | 1.00 |
| YLR263W | 1.00 |
| YGL164C | 1.00 |
| YDR410C | 1.00 |
| YCL001W | 1.00 |
| YHL030W | 1.00 |
| YOR184W | 1.00 |
| YCL039W | 1.00 |
| YHR124W | 1.01 |
| YLR427W | 1.01 |
| YMR155W | 1.01 |
| YMR302C | 1.01 |
| YGR044C | 1.01 |
| YOR023C | 1.01 |
| YER142C | 1.01 |
| YLR128W | 1.01 |
| YMR221C | 1.01 |
| YJL100W | 1.01 |
| YJR107W | 1.01 |
| YGR194C | 1.01 |
| YOR084W | 1.01 |
| YKL029C | 1.01 |
| YGR212W | 1.01 |
| YKR066C | 1.01 |
| YIR030C | 1.02 |
| YOR087W | 1.02 |
| YLR151C | 1.02 |
| YJR024C | 1.02 |
| YJR019C | 1.02 |
| YIR042C | 1.02 |

| | |
|---|---|
| YGR093W | 1.02 |
| YGR101W | 1.02 |
| YOR311C | 1.02 |
| YBR096W | 1.03 |
| YOR246C | 1.03 |
| YMR020W | 1.03 |
| YIR038C | 1.03 |
| YOR093C | 1.03 |
| YMR066W | 1.03 |
| YNR008W | 1.03 |
| YPL111W | 1.03 |
| YPL096W | 1.03 |
| YIR034C | 1.04 |
| YKL025C | 1.04 |
| YPL123C | 1.04 |
| YLR410W | 1.04 |
| YOR022C | 1.04 |
| YJR086W | 1.05 |
| YJR044C | 1.05 |
| YMR194C-B | 1.16 |
| YCR042C | |
| YMR122W-A | |
| YMR212C | |
| YFL036W | |
